# Phase Angle as Surrogate Marker of Muscle Weakness in Kidney Transplant Candidates Referred to Prehabilitation

**DOI:** 10.3390/nu16142245

**Published:** 2024-07-12

**Authors:** Ester Marco, María José Pérez-Sáez, Elena Muñoz-Redondo, Yulibeth G. Curbelo, Cindry Ramírez-Fuentes, Delky Meza-Valderrama, Carolina Acuña-Pardo, Mª Dolors Muns, Olga Vázquez-Ibar, Betty Odette Chamoun, Anna Faura-Vendrell, Anna Bach, Marta Crespo, Julio Pascual

**Affiliations:** 1 Physical Medicine and Rehabilitation Department, Hospital del Mar, 08003 Barcelona, Spain; emunozredondo@psmar.cat (E.M.-R.); yulibethgeraldine.curbelo.pena@psmar.cat (Y.G.C.); cramirez@psmar.cat (C.R.-F.); ccacunapardo@psmar.cat (C.A.-P.); 2 Rehabilitation Research Group, Hospital del Mar Research Institute (IMIM), 08003 Barcelona, Spain; delkymeza@gmail.com; 3 Faculty of Health and Life Sciences, Universitat Pompeu Fabra, Dr. Aiguader Building (Mar Campus), 08003 Barcelona, Spain; 4 Department of Nephrology, Hospital del Mar, 08003 Barcelona, Spain; mperezsaez@psmar.cat (M.J.P.-S.); bettyodette.chamoun.huacon@psmar.cat (B.O.C.); afaura@psmar.cat (A.F.-V.); abach@psmar.cat (A.B.); mcrespo@psmar.cat (M.C.); 5 Physical Medicine and Rehabilitation Department, National Institute of Physical Medicine and Rehabilitation (INMFRE), Panama City 0819, Panama; 6 Department of Endocrinology and Nutrition, Hospital del Mar, 08003 Barcelona, Spain; mmuns@psmar.cat; 7 Department of Geriatrics, Centre-Fòrum Hospital del Mar, 08019 Barcelona, Spain; ovazquez@psmar.cat; 8 Department of Nephrology, Hospital Universitario 12 de Octubre, 28041 Madrid, Spain; julpascual@gmail.com

**Keywords:** phase angle, muscle weakness, diagnostic accuracy, prehabilitation, advanced chronic kidney disease, kidney transplant

## Abstract

Phase angle (PhA), a marker of nutritional status obtained by bioelectrical impedance analysis (BIA), is associated with the integrity of cell membranes. Damage to muscle fiber membranes can impact muscle strength, which is related to adverse outcomes in adults with advanced chronic kidney disease (CKD). The main objective of this study was to determine the usefulness of the PhA in identifying muscle weakness in candidates for kidney transplants (KTs). Secondly, it aimed to examine the associations of PhA with other parameters of body composition, exercise performance, and muscle structure. Sensitivity, specificity, and area under the receiver operating characteristics curve were used to evaluate the PhA (index test) as a biomarker of muscle weakness. Muscle strength was estimated with maximal voluntary isometric contraction of the quadriceps (MVCI-Q) of the dominant side. Muscle weakness was defined as MVIC-Q < 40% of body weight. A total of 119 patients were evaluated (mean age 63.7 years, 75.6% men). A phase angle cut-off of 5.1° was identified to classify men with a higher likelihood of having low muscle strength in upper limbs (MVIC-Q 40% of their body weight). Male KT candidates with PhA < 5.1° had poorer exercise capacity, lower muscle strength, less muscle mass, and smaller muscle size. A PhA < 5.1° was significantly associated with an eight-fold higher muscle weakness risk (OR = 8.2, 95%CI 2.3–29.2) in a binary regression model adjusted by age, frailty, and hydration status. Remarkably, PhA is an easily obtainable objective parameter in CKD patients, requiring no volitional effort from the individual. The associations of PhA with aerobic capacity, physical activity, muscle mass, and muscle size underscore its clinical relevance and potential utility in the comprehensive evaluation of these patients.

## 1. Introduction

Individuals awaiting kidney transplants (KTs) frequently face restrictions in physical ability and exercise tolerance [1], which negatively affect their function, health-related quality of life (HRQoL), and clinical outcomes [2]. Impaired physical activity is linked to poorer post-KT outcomes, including diminished cardiorespiratory fitness, muscle strength, metabolic and nutritional deficiencies, as well as reduced quality of life and higher mortality rates [3,4]. The decline in functional capacity is due to various factors, such as aging, comorbidities, nutritional issues, and frailty [5]. Understanding the relationships between these factors and chronic diseases is crucial information for the clinical nutrition community and leading societies in clinical nutrition and metabolism.

Assessing body composition in patients with chronic kidney disease (CKD) presents considerable challenges. Various methods are available for muscle mass evaluation, from physical examination and anthropometric measurements to creatinine kinetic tests, bioelectrical impedance analysis (BIA), analysis of total body nitrogen by neutron activation, and imaging techniques such as dual-energy X-ray absorptiometry (DXA), computed tomography (CT), and magnetic resonance imaging (MRI) [6]. Although imaging methods are recognized as the most accurate for assessing muscle mass [7], bioimpedance techniques are more commonly applied in clinical settings [6].

The European Society for Clinical Nutrition and Metabolism (ESPEN) recommends prioritizing the use of quantitative methods (e.g., BIA) to assess muscle mass due to their reliability, non-invasiveness, cost-effectiveness, and practicality in both clinical practice and research [8]. In its guidance for the assessment of the muscle mass phenotypic criterion for the Global Leadership Initiative on Malnutrition (GLIM) diagnosis of malnutrition, the ESPEN highlights the growing recognition of the phase angle (PhA) as a potential marker of muscle mass [8]. PhA has been associated with cellular health, nutritional status, and exercise habits [9,10,11]. In healthy adults, normal PhA values typically range from 5° to 7°, with women generally having lower values than men, except in individuals over 70 years old [12]. PhA typically rises with body mass index (BMI) up to 35 kg/m^2^, after which it decreases again [12].

Muscle strength is a crucial health marker with significant prognostic implications in chronic diseases, as it reflects overall physical condition and functional capacity. Stronger muscle function is associated with better outcomes, including reduced risk of complications, improved quality of life, and lower mortality rates. Therefore, assessing muscle strength can provide valuable insights for the management and treatment of various chronic conditions. The assessment of muscle strength should be performed using dynamometry systems, which are standard in rehabilitation settings. However, in settings involving patients with advanced CKD, BIA serves as a practical method to detect patients with low muscle function. PhA is linearly related to muscle strength [13,14]. According to recent research, there is not only a positive correlation with muscle strength but also with skeletal muscle, gait speed, and various aspects of HRQoL in patients with CKD [14].

It is hypothesized that damage to the cell membrane of muscle fibers impacts their function. If this association is confirmed, determining PhA could be useful as a screening technique, either alone or in combination with other methods, for the early detection of individuals with muscle weakness.

Based on these considerations, the aim of this study was to evaluate the diagnostic efficacy of PhA in detecting individuals with decreased muscle strength during the prehabilitation of advanced CKD patients on the KT waiting list. The associations of PhA with other parameters of physical function, body composition, and muscle structure were also examined.

## 2. Materials and Methods

### 2.1. Study Design

This is a diagnostic accuracy study using data from a prospective cohort of KT candidates referred to a multimodal prehabilitation program. The study adheres to the Standards for Reporting of Diagnostic Accuracy Studies (STARD) guidelines [15].

### 2.2. Setting

The study was conducted in the Department of Physical Medicine and Rehabilitation at a tertiary university hospital.

### 2.3. Population, Eligibility Criteria

The cohort consisted of adults with advanced CKD awaiting deceased-donor kidney transplantation, who were directed to a prehabilitation program focused on enhancing functionality. Individuals with muscle-related conditions or injuries, as well as those unable to finish the exercise regimen, were not included. To conduct this diagnostic accuracy evaluation, two more criteria were enforced: finishing the initial cohort evaluation and having access to baseline BIA information.

### 2.4. Index Tests and Reference Standard

The index test was the measurement of PhA using the InBody S10^®^ system (Biospace, Cerritos, CA, USA) at a frequency of 50 Hz. For the test, patients lay supine on an examination table for 10 min, having removed all metallic objects. Tactile electrodes were attached to the thumbs and middle fingers of both hands, as well as beneath the malleoli of both ankles. Before placing the electrodes, a manufacturer-recommended wipe was used to enhance conductivity. Patients in hemodialysis were evaluated within the first 24 h after hemodialysis, and patients in peritoneal dialysis were evaluated within the first 8 h after peritoneal dialysis. However, this was not possible in all evaluations because of the patient’s compromised schedule. The patients were also advised to avoid drinking or eating anything two hours before the evaluation to prevent artefactual effects.

The reference test was the maximal voluntary isometric contraction (MVIC) of the quadriceps muscle on the dominant side. A high-resolution dynamometer (Mecmesin, Slinfold, UK) was used following a standardized procedure [16]. Patients sat on an examination table without a backrest, with their hands resting on their knees and the leg to be tested flexed at 90°. The highest value from three reproducible maneuvers was used; values below 40% of body weight were considered decreased.

Due to the post hoc nature of this study, the results of the reference test were not available to the investigator performing the index test. Similarly, of course, the index test measurements were not available to the reference test evaluators.

### 2.5. Other Study Variables

Estimates of body composition were derived using proprietary prediction models from the manufacturer of the In-Body S10^®^ impedance device (Biospace, Cerritos, CA, USA). These parameters included lean mass (in kg and as a percentage of reference population values), skeletal muscle mass (kg), and appendicular skeletal muscle mass index (AMMI) (kg/m^2^). Additionally, body composition parameters such as body fat (in kg and as a percentage of body weight), intra- and extracellular water (ICW and ECW, respectively), and total body water (TBW) were collected. The ECW/TBW ratio was also calculated, with a ratio greater than 0.390 indicating overhydration and less than 0.360 indicating dehydration.

Handgrip (HG) strength was measured using a digital dynamometer (Jamar Plus^®^, Nottinghamshire, UK), following the Southampton protocol [17,18]. The highest value from three reproducible maneuvers (each with less than 10% variability) was used for analysis. HG values were expressed in kg and as a percentage of the reference population; values <80% of the reference population indicated muscle weakness [19]. Respiratory muscle strength was evaluated using maximal inspiratory and expiratory pressures (PImax and PEmax, respectively). For analysis, the highest value from three reproducible maneuvers (with less than 10% variability between values) was considered. Predicted values based on a reference population were also calculated [20].

Exercise capacity was evaluated using maximal oxygen uptake (VO_2peak_) and workload (W_peak_) in a standardized incremental cardiopulmonary effort test (CPET) [21]. After 2 min of breathing at rest, subjects pedaled on an electrically braked cycle ergometer (Ergoline Ergoselect 4P, Bitz, Germany). An integrated computer system (Ergostik Cardiopart, Geratherm + Amedtec, Geratal, Germany) recorded cardiorespiratory variables throughout the test. Patients were encouraged to continue pedaling until they could no longer maintain the target pedaling frequency. Exercise capacity was also estimated with the distance traveled in a 6-minute walk test (6MWT) according to American Thoracic Society (ATS) guidelines [22]. Additionally, gait speed over a 4-m distance at a usual pace was recorded.

Ultrasound assessment of muscle size was performed following the SARCopenia measurement by UltraSound (SARCUS) group recommendations [23]. Muscle thickness was measured by ultrasound in both upper and lower limbs. For the upper limb, a cross-sectional view included the supinator, extensor carpi radialis brevis, extensor carpi radialis longus, and brachioradialis muscles. The probe was positioned at the proximal third of the bicipital tendon, extending to the radial styloid. The average of three reproducible measurements on the dominant forearm (provided it had no dialysis fistula) was used. A lower limb ultrasound was performed with the patient supine and legs extended. The probe was placed transversely at the midpoint between the greater trochanter and the superior pole of the patella. The thickness of the dominant rectus femoris muscle was measured from the inferior to the superior aponeurosis. The average of three consistent measurements from different images was recorded.

Potential confounding factors included modality of dialysis (peritoneal dialysis, hemodialysis, and non-kidney replacement therapy) and frailty, which was assessed using the Fried phenotype score [24]. This score includes unintentional weight loss (greater than 4.5 kg or more than 5% of body weight in the past year), reduced grip strength, decreased energy level (assessed using two questions from the Center for Epidemiologic Studies Depression Scale), slow walking speed, and low physical activity as measured by the short version of the Minnesota Leisure Time Physical Activity Questionnaire. Patients with a Fried score of three or higher were categorized as frail.

### 2.6. Ethics

We followed national and international ethical guidelines for research involving human subjects, including the Declaration of Helsinki and its amendments, as well as the Code of Ethics and Good Clinical Practice guidelines. Data management was conducted in compliance with current Spanish legislation and the General Data Protection Regulation (GDPR) of the European Union 2016/679, dated 27 April 2016. All participants received written information about the study procedures and provided signed informed consent. The study protocol and informed consent were reviewed and approved by the local Ethics Committee (No. 019/8623/I).

### 2.7. Statistical Analysis

Data were reported using means and standard deviations (SD) for quantitative variables and absolute values with percentages for categorical variables. Normality assumptions were assessed using the Kolmogorov–Smirnov test corrected with the Lilliefors test and normality plots. For univariate analyses, the Chi-square was used for categorical variables and the Student-t test for independent samples.

The diagnostic performance of the index tests was compared to the diagnostic criteria of EWGSOP2 (reference standard) [25] by calculating sensitivity, specificity, Youden index, positive negative value (PPV), negative predictive value (NPV), accuracy rate (proportion of true results for both index and reference tests), and likelihood ratios (LR+ and LR−). Validity thresholds were sensitivity and specificity >80% (indicating good validity), sensitivity or specificity <80% but both values >50% (indicating fair validity), and sensitivity or specificity <50% (indicating low validity) [26]. The reliability of muscle strength measurements, in relation to the EWGSOP criteria, was analyzed using contingency tables. Each 2 × 2 contingency table included two columns representing the EWGSOP criteria (yes or no) and one row for low or normal muscle strength. The area under the receiver operating characteristic curve (AUC) assessed the accuracy of muscle strength values in predicting sarcopenia. [27]; values nearing one were indicative of higher diagnostic accuracy [28]. The Youden index ranges from 0 to 1: a score of 1 indicates perfect sensitivity and specificity, whereas a score of 0 indicates that the index test is not useful.

Univariate and multivariate logistic regressions were conducted to identify covariates associated with a low PhA threshold. Crude or adjusted odds ratios (OR) with their corresponding 95% confidence intervals (95%CI) were reported. The multivariate analysis incorporated the significant variables in the univariate analysis and those deemed clinically relevant.

A post-hoc power calculation for both the AUC estimation and the results of the multivariate logistic regression was conducted. The analysis was conducted using IBM SPSS Statistics v.28 software (SPSS Inc., Chicago, IL, USA). The level of statistical significance was set at *p* ≤ 0.05. was used for analysis.

## 3. Results

**Figure 1:**
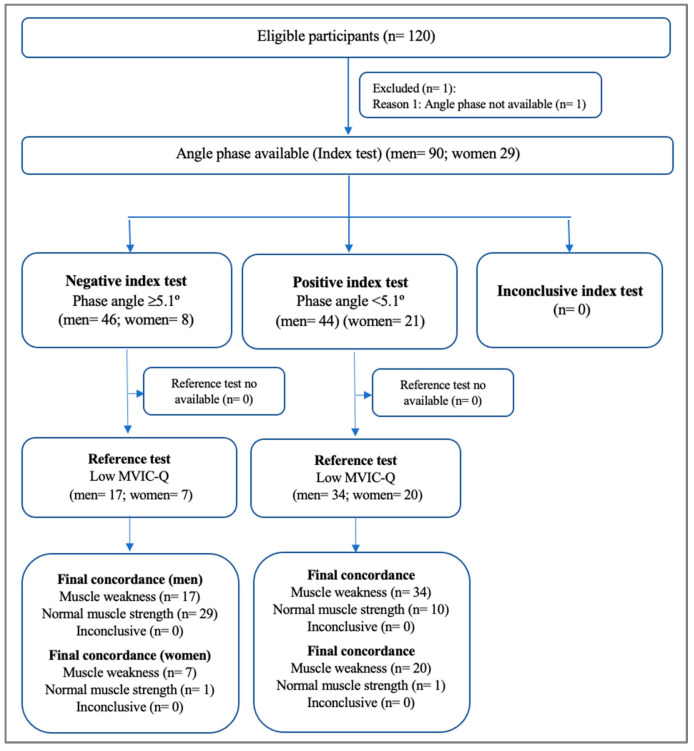
STARD diagram reporting flow of participants in the accuracy study. Abbreviation: MVIC-Q, maximal voluntary isometric contraction of the quadriceps muscle on the dominant side.

Figure 1 shows the STARD diagram reporting the flow of participants in the accuracy study. From the FRAILMar cohort data, 119 patients (mean age 63.7 years; 75.6% men) were evaluated. The demographic, clinical, and functional characteristics of the participants regarding muscle function, body composition, muscle size, and quality are shown in Table 1. Just over half of the patients were undergoing hemodialysis (54.6%), 19 (16%) were undergoing peritoneal dialysis, and 22 (18.5%) were not receiving any kidney replacement therapy. Sixty-four (53.8%) patients were prefrail or frail. Exercise capacity in terms of VO_2_ and workload were severely diminished (<50% pred.).

The prevalence of peripheral muscle weakness was 49.6% (*n* = 59) in upper limbs and 65.5% (*n* = 78) in lower limbs; maximal respiratory pressures were also under-predicted values. The mean value of PhA was 5.1° (SD 0.9) in men and 4.5° (SD 0.8) in women. Fat-free mass was within the range of normality for the European population [29]. Only 12 (13.3%) men and 3 (10.3%) women had percentages of body fat within the range of normality. The AEC/ACT ratio was 0.405 (DE 0.070), indicating edema; only 33 patients (27.7%) had normal hydration status.

**Table 1:** Baseline description of participants.

	Total Sample (*n* = 119)	Normality Range
Age (years)	63.7 (SD 10.6)	-
Sex, men (%)	90 (75.6%)	-
Dialysis modality, *n* (%): Hemodialysis Peritoneal dialysis peritoneal No kidney replacement therapy Unknown	65 (54.6%) 19 (16%) 22 (18.5%) 13 (10.9%)	-
Body mass index (Kg/m^2^)	28.4 (SD 5.3)	18.5–25 Kg/m^2^ [30]
Frail status, Fried phenotype 1–5 (%)	64 (53.8%)	0, robust; 0–1, pre-frail; 2–5, frail.
Gait speed (m/s)	1.1 (SD 0.3)	>1.2 m/s
Exercise capacity: Peak oxygen uptake (mL/min) ^b^ Peak oxygen uptake (% pred.) Peak workload (watts) ^b^ Peak workload (% pred.) Distance traveled in the 6-minute walking test (m) ^b^ Distance traveled in the 6-minute walking test (% pred.)	786.9 (SD 402.3) 42.6 (SD 17.3) 55.3 (SD 33.1) 38.6 (SD 20.8) 403.8 (SD 111.2) 86.8 (SD 21.3)	- >80% pred. - >80% pred. - >80% pred.
Muscle weakness: Upper limb (handgrip <80% ref.) Lower limb (quadriceps VMIC < 40% body weight)	59 (49.6%) 78 (65.5%)	- -
Dominant peripheral muscle strength: Handgrip (Kg) ^a^ Handgrip (% ref.) MVIC of quadriceps (Kg) MVIC of quadriceps (% body weight)	28.1 (SD 10.1) 80.5 (SD 23.4) 28.2 (SD 10.0) 36.3 (SD 10.8)	- 80–120% [19] - <40% of body weight
Respiratory muscle strength: Maximal inspiratory muscle pressure (cmH_2_O) ^b^ Maximal inspiratory muscle pressure (% pred.) Maximal expiratory muscle pressure (cmH_2_O) ^b^ Maximal expiratory muscle pressure (% pred.)	68.4 (SD 28.2) 63.8 (SD 23.9) 106.8 (SD 38.7) 63.8 (SD 20.5)	- >80% pred. - >80% pred.
BIA-derived parameters of body composition: Skeletal muscle mass (Kg) ^b^ Fat-free mass (Kg) ^b^ Fat mass (Kg) ^b^ Fat mass (% body weight) Total body water (L) ^b^ Extracellular water (L) ^b^ Intracellular water (L) ^b^ Extracellular water/Total body water ^b^ Phase angle (°)	27.7 (SD 6.0) 50.5 (SD 10.6) 27.2 (SD 12.2) 33.3 (SD 10.5) 37.5 (SD 7.7) 15.1 (SD 3.1) 22.8 (SD 4.6) 0.405 (SD 0.070) 4.95 (SD 0.9)	- - - Men 10–20%, women 18–28% [31] . - - 0.360–0.390 [32,33] 5°–7° [12]
Muscle size of the dominant side assessed by ultrasound: Forearm muscle thickness (mm) Rectus femoris muscle thickness (mm)	15.6 (SD 3.9) 17.8 (SD 4.3)	13.3–23.5 mm [34] Men 20–31 mm; women 16–24 mm [35]

^a^ Values vary according to age and sex; ^b^ values vary based on individual characteristics. Abbreviations: SD: standard deviation; MVIC, maximal voluntary isometric contraction; % ref.: percentage of reference values.

**Figure 2:**
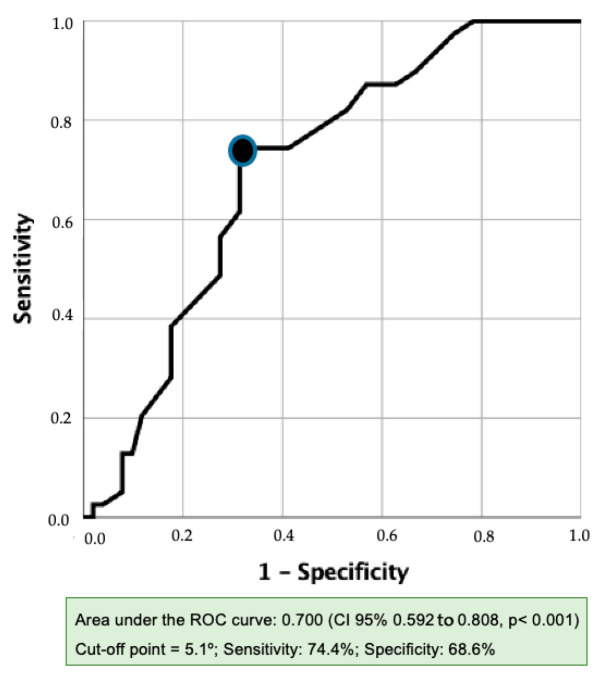
Receiver operating characteristic (ROC) curve for the detection of lower limb (maximal voluntary isometric contraction of the quadriceps) muscle weakness in men.

The determination of the PhA cut-off point for identifying muscle weakness was studied separately for men and women. However, since the cut-off point could only be established for men, the following data were generated by focusing on the analysis of the male patients in the cohort. Using the MVIC of quadriceps as the reference test, the area under the ROC curve for men was 0.700 (95%CI 0.592 to 0.808, *p* < 0.001) (Figure 2). A PhA threshold of ≤5.1° was determined using the maximum Youden index, with a sensitivity of 74.4% and a specificity of 68.6% (fair validity). A contingency table was constructed to show the frequency distribution of men with muscle weakness according to the PhA threshold of <5.1° (Table 2). The power calculation for the estimated area under the ROC curve (AUC = 0.700), having 51 weakness patients and 39 non-weakness patients, was 92.7%.

**Figure 3:**
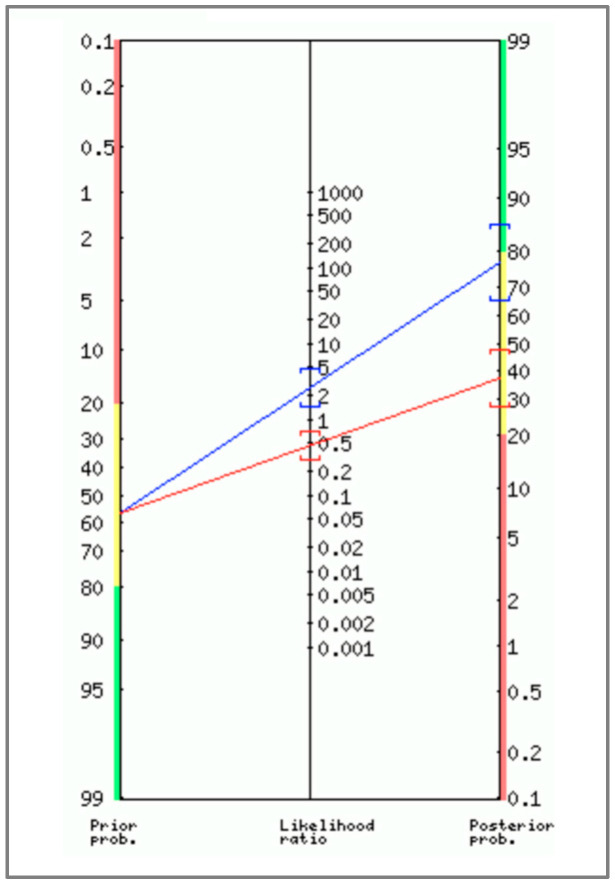
Positive and negative post-test probability of muscle weakness (blue line) and negative (red line) for upper limb (**left**) and lower limb (**right**) in male patients waiting for kidney transplantation. Nomograms were created using the ‘Diagnostic Test Calculator’ tool (available at: http://araw.mede.uic.edu/cgi-bin/testcalc.pl, accessed on 31 May 2024).

Table 3 summarizes the main diagnostic properties of PhA. Figure 3 shows the probabilities of a patient having reduced MVIC of quadriceps after a positive or negative test.

Bivariate analysis, based on the PhA cut-off point, detected statistically significant differences in age, gait speed, exercise capacity (peak oxygen uptake, peak workload, and distance traveled in the 6-min walking test), peripheral and respiratory muscle strength, appendicular skeletal muscle mass, and muscle size of upper and lower limbs (Table 4). In a multivariate logistic regression analysis, the crude OR for muscle weakness of the dominant quadriceps muscle was 5.8 (95%CI 2.3 to 14.6, *p* < 0.001), and the adjusted OR was 8.2 (95%CI 2.3 to 29.2, *p* = 0.001) after age, frailty, and hydration status adjustments (Table 5). Results from power calculation for the adjusted model showed a 94.9% power. There were no missing data, nor were there any reported adverse effects from the administration of the tests.

## 4. Discussion

This diagnostic accuracy study evaluates the values of the PhA and its potential usefulness as a marker for identifying muscle weakness in candidates for KT. The prevalence of muscle weakness was high (49.6% in upper limbs, 65.5% in lower limbs), similar to values reported in a cohort of 8767 patients with CKD of similar age and clinical characteristics [36]. BIA is a simple, non-invasive and low-cost technique that, like isometric dynamometry systems, can be made easily available in rehabilitation units. In addition, BIA and bioelectrical impedance spectroscopy (BIS) are commonly performed in dialysis centers for the estimation of body fluid. The PhA, a parameter based on reactance (Xc) and resistance (R), is a measure of how long the signal is delayed in Xc [37] and is easy to obtain in these patients. Reduced PhA values are associated with inflammation, cellular dysfunction, nutritional disorders, and mortality [38,39,40,41].

The ESPEN Guidance for the assessment of muscle mass highlights the PhA as a potential marker that deserves more research [8], but it is evident that exploring its relationship with muscle strength is of great interest to clinicians. In our sample, muscular weakness in the upper and lower limbs appears in male patients with a PhA below 5.1°.

The observed results are particularly noteworthy because they identify an objective parameter that requires no volition or active cooperation by the patient. This parameter is easily accessible in Nephrology and Rehabilitation settings. Moreover, we found correlations not only with the strength of peripheral and respiratory muscles but also with musculoskeletal mass, muscle size, walking speed, distance covered in the 6MWT-, and exercise capacity assessed with a CPET. Simply stated, this simple test can enable efficient identification of patients who will benefit the most from rehabilitation interventions.

Nonetheless, some limitations of the study should be taken into account when interpreting the results. First, the sample size was relatively small, and the limited sample size for women did not allow us to identify a PhA cut-off point for women. Second, we did not have access to the length of time participants had been on renal replacement therapy, which could influence their level of physical activity and functionality. Third, the potential for selection bias should also be considered, as patients with advanced CKD without KT criteria (e.g., severe disability or advanced dementia) were excluded. Finally, the timing of BIA measurement is a possible limitation. While limited research rigorously assesses the optimal timing for evaluating body composition in CKD patients, the most favorable timing seems to be when patients are near their dry weight. For those undergoing peritoneal dialysis, it is suggested to evaluate body composition when the abdominal cavity is empty [6]. In our sample, only 33 patients had normal hydration status (26.2% of hemodialysis patients, 21.1% of peritoneal dialysis patients, and 36.4% of non-dialysis patients). Despite being instructed to attend with an empty peritoneal cavity, many peritoneal dialysis patients were unable to comply due to the long distances they had to travel to the hospital for evaluations.

BIS and BIA, the preferred methods currently used to evaluate body composition in patients with advanced CKD [42,43], have some distinctive features. The InBody S10^®^ device (Biospace, Cerritos, CA, USA) uses six frequencies, while BIS uses a greater number of frequencies, which could provide better accuracy [44,45,46,47]. Another significant distinction is that fluid estimates by BIS are not based on empirical equations specific to a particular population. Instead, the Cole-Cole model is used to estimate fluid compartments, considering fat content, which is inversely related to ICW, ECW, and TBW [48,49]. In our study, we utilized the InBody S10^®^ device, which (unlike other BIA devices) employs the Cole-Cole method, similar to the BIS approach. This feature makes it an excellent tool for assessing body composition in patients with advanced CKD.

**Figure 4:**
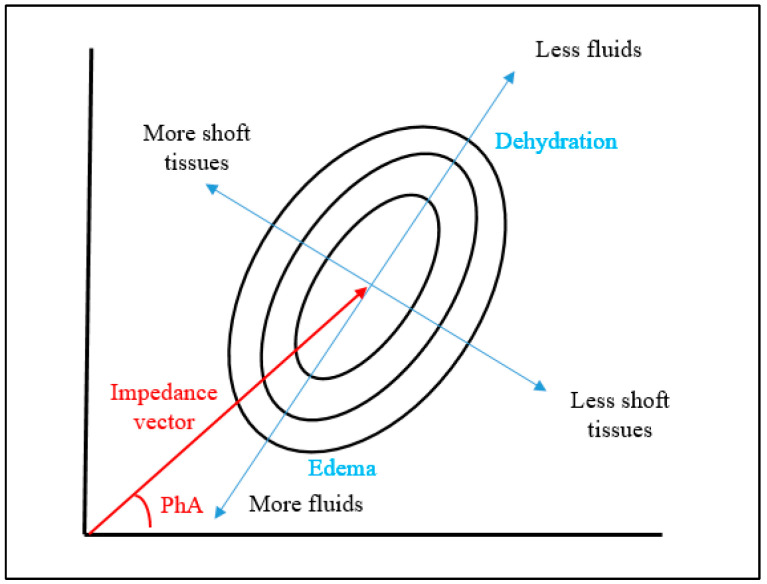
The Rx/Xc graph shows the impedance vector and phase angle (in red).

BIA does not replace the assessment of muscle strength using specific evaluation techniques such as dynamometry. However, it offers a non-invasive, quick, and cost-effective means to estimate muscle health, which is particularly useful in clinical settings where patients may have limited mobility or where access to specialized equipment is restricted. While dynamometry remains the gold standard for measuring muscle strength, BIA’s accessibility and ease of use make it a valuable alternative for regular monitoring in patients with CKD. The compilation of variations in altered fluid status (overload and dehydration), along with nutritional disorders, underscore the importance of the impedance vectors (Figure 4). While PhA focuses on phase and its relationship with body composition and cellular health, the vector in the R/Xc graph adds an additional dimension by showing how resistance (R) and reactance (Xc) vary together, providing a more comprehensive graphical representation of body composition and fluid distribution [50]. By using these parameters, clinicians can obtain important insights into muscle health and overall condition without the need for extensive resources, thus supporting its integration into routine practice for the management of CKD patients.

## 5. Conclusions

In summary, muscle weakness appears to be prevalent among candidates for KT. A PhA cut-off of 5.1° was identified to classify men with a higher likelihood of having low muscle strength in upper limbs (sensitivity 82.4%, specificity 60.9%). This threshold was associated with slower gait speed, poorer exercise capacity, lower muscle strength, lesser muscle mass, and smaller muscle size. These associations underscore its clinical relevance and potential utility in the comprehensive evaluation of these patients. In men, PhA values below 5.1° are significantly associated with an eight-fold higher risk of muscle weakness (OR = 8.2, 95%CI 2.3–29.2) in a binary regression model adjusted by age, frailty, and hydration status. It is important to clarify that these results are applicable specifically to men. Further research in a larger sample will be required to validate the utility of PhA measurement in detecting muscle weakness in women and other populations. Extrapolating these findings to different demographic groups will require broader and more specific analysis to determine their applicability and accuracy in those particular contexts.

## Figures and Tables

**Table 2:** Frequency distribution of phase angle (cut-off point <5.1°) and muscle weakness of lower limbs in men on the kidney transplant waiting list referred to prehabilitation.

	Reference Test (MVIC-Q < 40% of Body Weight)			
		Weakness (*n* = 78)	No Weakness (*n* = 41)	Total (*n* = 119)
Phase Angle < 5.1°	Positive	37	28	65
	Negative	22	32	54

Abbreviations: MVIC-Q, maximal voluntary isometric contraction of the quadriceps muscle on the dominant side.

**Table 3:** Diagnostic ability of the index test (phase angle <5.1°) for detecting lower limb weakness in men on the kidney transplant waiting list referred to prehabilitation.

	MVIC of Quadriceps (<40% of Body Weight)
Sensitivity	66.7%
Specificity	74.4%
Positive predictive value	77.3%
Negative predictive value	63.0%
Accuracy	70%
Positive likelihood ratio	2.6
Negative likelihood ratio	0.45

Abbreviations: MVIC, maximal voluntary isometric contraction.

**Table 4:** Bivariate analysis of phase angle values (<5.1°) in male candidates for kidney transplant (*n* = 90).

	PhA ≥ 5.1° (*n* = 46)	PhA < 5.1° (*n* = 44)	Mean Differences (95%CI)	*p*
Age (years)	60.1 (SD 10.9)	67.2 (SD 8.7)	7.2 (3.1 to 11.3)	0.001
Dialysis modality, *n* (%) Hemodialysis Peritoneal dialysis No kidney replacement therapy Information not available	26 (56.5%) 6 (13.0%) 9 (19.6%) 5 (10.9%)	28 (63.6%) 7 (15.9%) 4 (9.1%) 5 (11.4%)	-	0.363
Body mass index (Kg/m^2^)	28.3 (SD 4.8)	27.5 (SD 4.9)	−0.8 (−2.8 to 1.2)	0.435
Frailty, Fried phenotype 3–5 (%)	6 (13.0%)	9 (20.5%)	-	0.405
Gait speed (m/s)	1.2 (SD 0.2)	1.0 (SD 0.2)	−0.2 (−0.3 to −0.1)	0.001
Exercise capacity: Peak oxygen uptake (L/min) Peak oxygen uptake (% pred.) Peak workload (watts) Peak workload (% pred.) Distance traveled in the 6-minute walking test (m) Distance traveled in the 6-minute walking test (% pred.)	1.0 (SD0.5) 47.0 (SD 17.8) 71.8 (SD 34.2) 45.0 (SD 18.2) 471.7 (SD 103.4) 95.7 (SD 17.2)	0.7 (SD 0.3) 37.2 (SD 15.9) 50.7 (SD 29.9) 36.7 (SD 24.1) 359.4 (SD 88.7) 79.9 (SD 22.3)	−0.3 (−0.5 to −0.1) −9.7 (−17.6 to −1.9) −21.1 (−35.6 to −6.6) −8.3 (−17.8 to 1.2) −112.4 (−153.4 to −71.4) −15.8 (−24.2 to −7.4)	0.001 0.016 0.005 0.086 <0.001 <0.001
Peripheral muscle strength of the dominant side: Handgrip (Kg) Handgrip (% ref.) MVIC of quadriceps (Kg) MVIC of quadriceps (% body weight)	35.3 (SD 9.5) 84.4 (SD 24.1) 34.7 (SD 10.0) 42.9 (SD 9.4)	27.8 (SD 5.5) 77.6 (SD 23.2) 27.1 (SD 7.3) 34.7 (SD 9.1)	−7.5 (−10.7 to −4.2) −6.8 (−16.7 to 3.1) −7.6 (−11.3 to −3.9) −8.2 (−12.1 to −4.4)	<0.001 0.174 <0.001 <0.001
Respiratory muscle strength: Maximal inspiratory muscle pressure (cmH_2_O) Maximal inspiratory muscle pressure (% pred.) Maximal expiratory muscle pressure (cmH_2_O) Maximal expiratory muscle pressure (% pred.)	81.0 (SD 30.8) 68.3 (SD 24.9) 123.4 (SD 42.7) 66.3 (SD 21.9)	64.0 (SD 24.2) 58.4 (SD 22.8) 104.0 (SD 31.8) 59.7 (SD 18.7)	−17.0 (−28.7 to −5.4) −9.9 (−19.8 to 0.2) −19.2 (−35.2 to −3.5) −6.6 (−15.1 to 1.9)	0.005 0.054 0.017 0.128
BIA-derived parameters of body composition: Appendicular skeletal muscle mass (Kg) Fat-free mass (Kg) Fat mass (Kg) Fat mass (% body weight) Extracellular water / Total body water	31.1 (SD 5.4) 54.9 (SD 11.3) 25.6 (DE 12.7) 29.0 (SD 8.8) 0.404 (SD 0.11)	28.0 (DE 4.4) 52.1 (SD 7.5) 26.7 (SD 12.8) 32.5 (SD 10.5) 0.409 (SD 0.03)	−3.2(−5.2 to 1.1) −2.8 (−6.8 to 1.2) 1.1 (−4.2 to 6.5) 3.5 (−0.5 to 7.6) 0.055 (−0.03 to 0.04)	0.003 0.168 0.678 0.086 0.761
Muscle size assessed by ultrasound: Dominant forearm muscle thickness (mm) Dominant rectus femoris muscle thickness (mm)	17.6 (SD 3.8) 21.0 (SD 3.4)	15.2 (SD 3.6) 16.3 (SD 4.0)	−2.3 (−3.9 to −0.8) −4.7 (−6.2 to −3.1)	0.004 <0.001

Note: Student t-test for independent variables was used to compare quantitative variables, and Chi-squared or Fisher exact test for categorical variables. Abbreviations: BIA, bioimpedance analysis; MVIC, voluntary maximal isometric contraction; PhA, phase angle; % ref.: reference value percentage.

**Table 5:** Muscle weakness Odds ratios for phase angle <5.1° (dependent variable) adjusted by frailty, age, and hydration status (in men). Raw and adjusted results.

	Crude Analysis (Univariate)			Multivariate Analysis		
Muscle Weakness	cOR	95%CI	*p*	aOR	95%CI	*p*
Phase angle (<5.1°)	5.8	2.3 to 14.6	<0.001	8.2	2.3 to 29.2	0.001
Age				1.0	1.0 to 1.1	0.980
Frailty				0.9	0.3 to 3.2	0.899
Hydration status				0.6	0.2 to 2.3	0.586

Abbreviations: cOR, crude odds ratio; 95%CI, 95% confidence interval; aOR, adjusted odds ratio.

## Data Availability

The data are not publicly available because the Frailmar study has not yet concluded. Data are available upon reasonable request to the corresponding author.
